# *In silico* and *in vitro* analyses for the improved diagnosis of bacterial meningitis

**DOI:** 10.3389/fmicb.2025.1655490

**Published:** 2025-09-26

**Authors:** Simon T. L. Amoikon, Kanny Diallo, Jeremie K. Tuo, Naima Nasir, Vitalis F. Feteh, Grace Mzumara, Adeniyi Aderoba, Rebecca Jacques, Hansini Mandal, Keith A. Jolley, James E. Bray, Odile B. Harrison, Martin C. J. Maiden

**Affiliations:** ^1^Centre Suisse de Recherches Scientifiques en Côte d’Ivoire (CSRS), Abidjan, Côte d’Ivoire; ^2^Institut Pierre Richet (IPR), Institut national de santé publique (INSP), Bouaké, Côte d’Ivoire; ^3^Institut National Polytechnique Felix Houphouët-Boigny, Yamoussoukro, Côte d’Ivoire; ^4^Department of Biology, University of Oxford, Oxford, United Kingdom; ^5^Nuffield Department of Population Health, University of Oxford, Oxford, United Kingdom

**Keywords:** meningitis, molecular diagnostic, *in silico* analysis, sensitivity, specificity

## Abstract

**Context:**

Diagnosing meningitis remains challenging with etiological agents frequently unidentified. Using both *in silico* and *in vitro* approaches, this study evaluated published and novel genetic targets for the detection of common bacterial species known to cause meningitis: *Neisseria meningitidis*, *Streptococcus agalactiae*, *Streptococcus pneumoniae*, and *Haemophilus influenzae*.

**Methods:**

A total of 29 genetic targets were investigated for the detection of *N. meningitidis*, *S. agalactiae*, *S. pneumoniae*, and *H. influenzae*, using the Gene Presence tool and whole genome sequence data (WGS) found in the genomics platform, PubMLST. These targets were further tested *in silico* by screening WGS using the PCR tool hosted on PubMLST allowing the sensitivity, specificity, Negative Predicted Values (NPV) and Positive Predictive Values (PPV) to be determined. Ten targets were then further evaluated *in vitro* by real-time PCR against a panel of 44 bacterial isolates representative of the genera evaluated.

**Results:**

The best performing *in silico* genetic determinants targeted: *N. meningitidis, sodC* (NEIS1339) (sensitivity 99.7%, specificity, 99.4%, PPV, 99.6% and NPV, 99.6%)*; S. pneumoniae, SP2020* (99.5%, 99.9%, 99.9%, and 81.5%) and *H. influenzae*, *dmsA* (HAEM1183) (98%, 100%, 99.6%, and 77.4%). All three of these targets also had the best *in vitro* sensitivity (100%), specificity [91.7% *sodC* (NEIS1339), 100% *SP2020* and 97.3% *dmsA* (HAEM1183), PPV (72.7% *sodC* (NEIS1339), 100% *SP2020* and 87.5% *dmsA* (HAEM1183)] and NPV (100% for all targets). The gene *sip* (SAG0032) encoding the surface immunogenic protein (*sip*) exhibited the best sensitivity (99.6%) and NPV (96.9%) for *S. agalactiae* compared to 99.3% and 94.8% for *cfb* (SAG2043), respectively *in silico*. However, *in vitro*, *cfb* showed the best sensitivity (100% vs. 85.7%) and NPV (100% vs. 97.4%) when compared to *sip*.

**Conclusion:**

*SodC, cfb, SP2020*, and *dmsA* have the potential to enhance the accuracy of molecular diagnostics for the four most common bacterial species causing meningitis. Moreover, a combined *in silico* and *in vitro* approach that leverages WGS deposited in databases such as PubMLST, offers an efficient and cost-effective means for the preliminary evaluation of diagnostic targets.

## 1 Introduction

Bacterial meningitis and related invasive infections, including pneumonia, bacteraemia and septicaemia, are devastating diseases that represent a major public health concern worldwide (Rodgers et al., 2020), the main bacterial aetiological agents being: *Neisseria meningitidis* (the meningococcus); *Streptococcus pneumoniae* (the pneumococcus); *Haemophilus influenzae*; and *Streptococcus agalactiae* (group B streptococcus, GBS). Despite the availability of vaccines against many variants of these pathogens (Tsang, 2021), with the exception of *S. agalactiae* for which intrapartum antibiotic prophylaxis is used (Hughes et al., 2017), meningitis continues to affect populations globally, particularly in sub-Saharan Africa.

In sub-Saharan Africa, meningitis is endemic, with seasonal epidemics occurring unpredictably every 5–12 years (Hughes et al., 2017). A recent example being the meningitis outbreak among miners in the northeastern cities of the Democratic Republic of Congo, declared on 8 September 2021 (WHO Africa, 2022). These epidemics are associated with a high case fatality rate of 50% and many cases remain undiagnosed, increasing the delay in appropriate public health interventions. Control of meningitis requires improved access to and efficiency of diagnostic methods; indeed, diagnosis is one of the five pillars of the World Health Organisation (WHO) roadmap to defeating meningitis by 2030 (World Health Organization [WHO], 2021).

Efficient surveillance, outbreak investigation and clinical management of meningitis depends on laboratory confirmation of the causative pathogen from sterile sites, such as cerebrospinal fluid (CSF) and blood. The “gold standard” methods for confirmation of meningitis remain: (i) culture and (ii) polymerase chain reaction (PCR). Culture allows an isolate to be retained for further use; however, this can take at least 24 h and is often more challenging in sub-Saharan Africa due to long transportation time and/or previous antimicrobial treatment (Diallo et al., 2021). PCR is a rapid molecular diagnostic method that enables identification within a few hours. While PCR is sensitive, specific and does not depend on the presence of viable bacteria, it requires expensive equipment, reagents and expertise (Diallo et al., 2021; Feagins et al., 2020; Griffiths et al., 2018). The success of PCR depends on the presence of genomic regions specific to each pathogen. The genetic diversity of meningitis-associated pathogens (Spratt and Maiden, 1999) and their genomic variability indicates there is an on-going need to monitor the effectiveness of existing molecular diagnostic tests targeting pathogens associated with meningitis. It is also important to continue searching for novel genetic targets that are more sensitive and specific than those currently used, while also considering their genetic variability.

A narrative review identified 25 genetic targets used in the detection of *H. influenzae, N. meningitidis, S. pneumoniae*, and *S. agalactiae* (Diallo et al., 2021). Testing these targets *in vitro* is costly and time-consuming. An alternative is to identify suitable targets *in silico* through bioinformatic analyses using large genome datasets and then confirming their appropriateness *in vitro* using a reduced panel of bacterial isolates (van Weezep et al., 2019).

The development of high-throughput whole genome sequencing has led to the creation of genome databases such as PubMLST, which contain bacterial population sequence data and provenance metadata for over 100 species and genera (Jolley et al., 2018). This platform receives thousands of yearly submissions including new sequences, allele profiles and isolate records (Jolley et al., 2018). *N. meningitidis, S. pneumoniae, H. influenzae*, and *S. agalactiae* WGS deposited in PubMLST include data from healthy carriers, invasive disease cases and other clinical sources.

This study aimed to evaluate *in silico* published genetic targets used in PCR assays for the detection of *N. meningitidis, S. pneumoniae, H. influenzae*, and *S. agalactiae* and identify optimal genetic targets. These were then tested *in vitro* using a panel of bacteria strains representative of the genera present.

## 2 Materials and methods

### 2.1 *In silico* analyses

#### 2.1.1 Whole genome sequence data (WGS)

*In silico* analyses were performed on WGS belonging to 70,697 isolate records stored in PubMLST^1^ (Jolley et al., 2018): 1964 *H. influenzae* (*Hi*); 146 WGS from other *Haemophilus* species (non-*Hi*); 8,793 *S. agalactiae* (GBS); 1,181 WGS from other streptococci including 463 *S. pneumoniae* (non-GBS); 14,401 *N. meningitidis* (*Nm*); 10,186 from other *Neisseria* species (non-Nm); 33,267 of *S. pneumoniae* (*Sp*); and 761 WGS from other streptococci including 44 *S. agalactiae* (non-*Sp*). The average genome lengths were: *H. influenzae*, 1.8 Mb, *S. agalactiae*, 2.2 Mb; *N. meningitidis*, 2.1 Mb; and *S. pneumoniae*, 2.1 Mb, with contig lengths averaging 400 bp.

A library of type strain genomes (*n* = 18,500) was annotated in the PubMLST Ribosomal MLST database^2^ (Jolley et al., 2012) to provide a comprehensive reference for species identification and facilitate accurate genome comparisons. This extensive library enables standardized comparisons between query genomes and a well-defined set of reference genomes, minimizing ambiguity during species identification. It complements species-specific databases by allowing efficient identification of unknown or mixed-species samples. To facilitate efficient genome comparison, the FastANI program (Jain et al., 2018) was employed to scan query genomes against this library. Initially, the MASH algorithm (Ondov et al., 2016) was utilized to identify the 10 nearest type strains. Subsequently, FastANI was applied to this subset to calculate the Average Nucleotide Identity (ANI) values. The type strain genome exhibiting the highest identity percentage was reported. While it is possible that the query genome may contain two or more species, only the top match was documented.

The *in silico* PCR, Gene Presence, and Field Breakdown plugins of the BIGSdb software (Jolley and Maiden, 2010) were used to analyze WGS data. *In silico* PCR was performed with a stringent criterion of no-mismatch for all sets of primers (primer sequences are listed in Supplementary Table 1) and the results were used to calculate the specificity, sensitivity, positive predictive value (PPV) and negative predictive value (NPV) for each assay. The Gene Presence tool, using default settings, was used to detect whole genome sequence data lacking any of the genes examined (genes analyzed are listed in Supplementary Table 1). This was undertaken in each pathogen-specific database using annotated full coding sequences of genes of interest as defined in PubMLST. A low detection rate despite high gene presence was taken as evidence of sequence divergence at primer binding sites. Only targets with consistent detection were retained. The Field Breakdown tool was used to assess results in association with available metadata with a focus on clinical sources, i.e., bacteremia, meningitis, other invasive diseases, carriage or not specified. We defined the best target as one that exhibited the highest sensitivity and specificity, detected in the majority of WGS. All genes defined in the PubMLST database are assigned a unique locus name, starting with “HAEM” for *Haemophilus*, “NEIS” for *Neisseria*, “SPNE” for *S. pneumoniae*, and “SAG” for *S. agalactiae*, followed by an arbitrary number. Additionally, each locus may also be associated with a common gene name. For example, NEIS1339 corresponds to the *sodC* gene.

In PubMLST there are multiple fields that can provide information on the bacterial capsule type: the isolates fields including for *Nm*, “serogroup” and “capsule group,” for GBS, “capsular serotype,” for Hi “serotype” and for Sp “submitted serotype” are filled by the submitter based on confirmatory tests done in their lab, serological or PCR tests. When genomes are available, the fields “genogroup” for *Nm* or “genotype,” “capsular genotype” for GBS, “genotype” for Hi and “serotype” for *Sp* indicate the capsule type based on the analysis of the *cps* genes involved in capsule synthesis, identified through the submitted whole genome sequences. The analysis of serotype/genotype done in the study were based on the genomic typing fields for all four pathogens. Isolates were categorized by PubMLST as non-typeable (NT) when they had non-functional or absent capsule genes (for example, *Haemophilus influenzae* non-typeable strains where the cps genes are absent). In the occasion were the genes were truncated at the end of a contig, the isolates were classified as undetermined, indicating that serotypes’ assignment could not be done.

#### 2.1.2 Identification of improved targets for *H. influenzae* detection

The Genome Comparator tool (Jolley and Maiden, 2010) in the PubMLST database^3^ was used to identify improved genetic targets for the detection of *H. influenzae* that achieved a sensitivity greater than 96.3% and a specificity greater than 94.7% compared with published genetic targets (Diallo et al., 2021). Genome Comparator analysis was conducted using the 1,898 loci defined in the database with a set core presence threshold of 97%, meaning that only genes present in at least 97% of *H. influenzae* WGS were considered for further analyses. Pairwise allelic differences between isolates were calculated using default settings: a minimum sequence identity of 70%, a minimum alignment coverage of 50%, and a BLASTN word size of 20. Nucleotide sequences of identified loci were compared to sequences deposited in GenBank using the BLAST program (Altschul et al., 1990) to confirm species specificity. In parallel, a BLAST search of targets identified from the Genome Comparator analysis, was undertaken in a collection of non *influenzae Haemophilus* genomes (non-Hi), using WGS stored in the Ribosomal Multilocus Sequence Typing (rMLST: see text footnote 2) database (Jolley et al., 2012) to confirm the absence of those targets in non-Hi species. A gene was considered absent if the length of the aligned sequence was less than half of the total length of the sequence of that gene. Primers and probes were designed for each selected target using Primer 3 (Untergasser et al., 2012) with default settings and tested *in silico* before applying *in vitro*.

### 2.2 *In vitro* analyses

#### 2.2.1 Bacterial strains and growth conditions

Reference strains were obtained from the National Collection of Type Cultures (NCTC). These were *H. influenzae* NCTC8143, *Haemophilus aegyptius* NCTC8502, *Haemophilus haemolyticus* NCTC10659, *S. pneumoniae* NCTC7465, *S. agalactiae* NCTC8181, *Streptococcus mitis* NCTC12261 and *Neisseria lactamica* NCTC10617 (Supplementary Table 2). *H. influenzae* NCTC8143, *H. aegyptius* NCTC8502 and *H. haemolyticus* NCTC10659 were cultured on Chocolate agar plate with sheep blood with the remaining species cultured on blood agar with sheep blood. Plates were incubated at 37 °C in 5% CO_2_ for 24 h.

#### 2.2.2 DNA samples

A total of 44 DNA samples were used for real-time PCR assays (Supplementary Table 2). Seven DNA samples were extracted from the reference strains using the Wizard^®^ Genomic DNA Purification Kit, following manufacturer’s instructions (Promega, United States). Twenty-eight additional DNA samples extracted from pure cultures of *H. influenzae, S. pneumoniae, S. agalactiae*, *N. meningitidis, N. gonorrhoeae, N. lactamica*, and *H. haemolyticus*, were kindly donated by Dr. Mignon du Plessis from the National Institute for Communicable Diseases of South Africa for this study. Among these 28 DNA extracts, four were from control strains and the others from specimens isolated from blood cultures, CSF, pleural fluid and patient tissue (Supplementary Table 2). In addition, nine DNA samples extracted from *N. lactamica*, *Neisseria sp., N. meningitidis*, and *Moraxella catarrhalis* isolates from a collection at Centre Suisse de Recherches Scientifiques in Côte d’Ivoire (CSRS) were used. These have been cultured from oropharyngeal swabs and saliva samples collected from healthy carriers as part of a carriage study conducted in a cohort of school children in Côte d’Ivoire (Missa et al., 2024) and their identity confirmed by WGS (Data not shown).

#### 2.2.3 Real-time PCR

Real-time PCR amplifications were carried out targeting the two genetic determinants most prevalent in WGS and exhibiting the highest sensitivities and specificities with at least 95% identified in in silico analyses (top two best target genes). In cases where the PCR did not work, a third gene, was tested (Table 1). For H. influenzae, two additional high-scoring genes were also tested. Assays were performed on a CFX96 Touch™ Real-Time PCR Detection system (Bio-Rad) using the TaqMan^®^ Gene Expression Master Mix (Applied Biosystems). The reaction mixture consisted of 7.5 μL of 2× Master Mix, 0.5 μM of each primer (forward and reverse), 0.5 μM of probe (Table 1), template DNA (2 μL) and UltraPure DNase/RNase-Free Distilled Water for a final volume of 15 μL. Positive controls and no-template control were included in each experiment. The cycling parameters consisted of 2 min at 50 °C, 10 min at 95 °C, 45 cycles of 95 °C for 15 s and 60 °C for 1 min, and then a holding stage at 4 °C. Samples with Ct values below 35 were considered positive, those above 40 negative, and values between 35 and 40 were classified as equivocal, unless otherwise specified. Equivocal samples were diluted 1:10 to reduce potential inhibitors and retested (Pouladfar et al., 2022).

**TABLE 1:** Primer sequences used for real-time polymerase chain reaction (PCR).

Gene	Primer name^a^	5′–3′ nucleotide sequence	Observation	References
*fucK*	fucK-F	ATGGCGGGAACATCAATGA	Not worked using published conditions	Meyler et al., 2012
	fucK-R	ACGCATAGGAGGGAAATGGTT		
	fucK-PB	FAM-CGGTAATTGGGATCCAT-MGB		
hpd	hpdF822	GGTTAAATATGCCGATGGTGTTG	Worked using published conditions	Wang et al., 2011
	hpdR952	TGCATCTTTACGCACGGTGTA		
	hpdPb896i1^b^	FAM-TTGTGTACACTCCGTTGGTAAAAGAACTTGCAC-BHQ		
pstA	pstA-F	CGTTTCGCACAAATTACC	Worked using published conditions	Coughlan et al., 2015
	pstA-R	GTGCGTACCACGATAGG		
	pstA-PB	FAM-CTGGAGCATTCGCATTAGCTT-BHQ		
cfb	cfb-F2	GAAACATTGATTGCCCAGC	Worked using published conditions	Carrillo-Ávila et al., 2018
	cfb-R2	AGGAAGATTTATCGCACCTG		
	cfb-PB2	Cy3-CCATTTGATAGACGTTCGTGAAGAG-BHQ		
dltS	dltS-F3	CCTTATGGCGTTCCACGATT	Not worked using published conditions	Furfaro et al., 2017
	dltS-R3	ATCATGCAGATTCTCTCAGTTTTGG		
	dltS-PB3	Cy3-CCTTAGCAATAGATAAGCCTAG-BHQ		
Sip	sip-F	ATCCTGAGACAACACTGACA	Worked using published conditions	Bergh et al., 2004
	sip-R	TTGCTGGTGTTTCTATTTTCA		
	sip-PB	Cy3-ATCAGAAGAGTCATACTGCCACTTC-BHQ		
sodC	F351	GCACACTTAGGTGATTTACCTGCAT	Worked using published conditions	Thomas et al., 2011
	R478	CCACCCGTGTGGATCATAATAGA		
	Pb387	JOE-CATGATGGCACAGCAACAAATCCTGTTT-BHQ		
porA	porA_fwd_1	GCCGGCGTTGATTATGATTT	Worked using published conditions	Diallo et al., 2018
	porA_rev_1	AGTTGCCGATGCCGGTATT		
	porA_pb_1	JOE-CTTCCGCCATCGTGTC-BHQ		
psaA	psaA forward	GCCCTAATAAATTGGAGGATCTAATGA	Worked using published conditions	Carvalho et al., 2007
	psaA reverse	GACCAGAAGTTGTATCTTTTTTTCCG		
	psaA probe1^b^	Cy5-CTAGCACATGCTACAAGAATGATTGCAGAAAGAAA-BHQ		
SP2020	SP_2020_F	TAAACAGTTTGCCTGTAGTCG	Worked using published conditions	Tavares et al., 2019
	SP_2020_R	CCCGGATATCTCTTTCTGGA		
	SP_2020_P	Cy5-AACCTTTGTTCTCTCTCGTGGCAGCTCAA-BHQ		
HAEM0428	HAEM0428_F	TGCCTGTATTTTAGCGATCCG	This study	This study
	HAEM0428_R	ATTAGCCTCAATGATCGCCG		
	HAEM0428_PB	FAM-CTGTTGTCCATTGCCCATGT-BHQ1		
HAEM1183	HAEM1183_F	TATGGTACGGGAACACTCGG	This study	This study
	HAEM1183_R	ATTTCCCAATGCCCAACCAC		
	HAEM1183_PB	FAM-GTGATTACAGCACCGCACAA-BHQ1		

^a^All fluorophores and quenchers of probes have been modified from what has been published with the exception of fucK and pstA fluorophores and SP2020 quencher.

^b^The quencher that was internal has been moved to the 3′ end.

### 2.3 Statistical analysis

The sensitivity and specificity of assays were determined using the following formula:


$$Sensitivity\left(\%\right)=\frac{T\,r\,u\,e\,p\,o\,s\,i\,t\,i\,v\,e\times 100}{T\,r\,u\,e\,p\,o\,s\,i\,t\,i\,v\,e+f\,a\,l\,s\,e\,n\,e\,g\,a\,t\,i\,v\,e}$$

and


$$Specificity\left(\%\right)=\frac{T\,r\,u\,e\,n\,e\,g\,a\,t\,i\,v\,e\times 100}{T\,r\,u\,e\,n\,e\,g\,a\,t\,i\,v\,e+f\,a\,l\,s\,e\,p\,o\,s\,i\,t\,i\,v\,e}$$

Positive predictive value (PPV) and negative predictive value (NPV) were determined according to the following formula:


$$PPV\left(\%\right)=\frac{T\,r\,u\,e\,p\,o\,s\,i\,t\,i\,v\,e\times 100}{T\,r\,u\,e\,p\,o\,s\,i\,t\,i\,v\,e+f\,a\,l\,s\,e\,p\,o\,s\,i\,t\,i\,v\,e}$$

and


$$NPV\left(\%\right)=\frac{T\,r\,u\,e\,n\,e\,g\,a\,t\,i\,v\,e\times 100}{T\,r\,u\,e\,n\,e\,g\,a\,t\,i\,v\,e+f\,a\,l\,s\,e\,n\,e\,g\,a\,t\,i\,v\,e}$$

## 3 Results

### 3.1 Geographic and temporal distribution of datasets

*H. influenzae* sequences dated from 1941 to 2020 and originated from Europe (917/1964, 46.7%), North America (805/1964, 41%), Oceania (115/1964, 5.9%), Africa (83/1964, 4.2%), Asia (32/1964, 1.6%), Unknown (10/1964, 0.5%), and South America (2/1964, 0.1%). *S. agalactiae* sequences dated from 1953 to 2018 and originated from North America (4,881/8793, 55.5%), Europe (1,652/8793, 18.8%), Africa (449/8793, 16.5%), Unknow (389/8793, 4.4%), Asia (239/8793, 2.7%), Oceania (174/8793, 2%), and South America (9/8793, 0.1%). *N. meningitidis* sequences dated from 1915 to 2021 and originated from Europe (9,872/14,401, 68.5%), North America (1,927/14,401, 13.4%), Africa (1,350/14,401, 9.4%), Asia (617/14,401, 4.3%), South America (244/14,401, 1.7%), Oceania (377/14,401, 2.6%) and Unknown (14/14,401, 0.1%). *S. pneumoniae* sequences dated from 1916 to 2018 and originated from Africa (10,754/33,267, 32.3%), Europe (8,274/33,267, 24.9%), Asia (7,621/33,267, 22.9%), North America (4,785/33,267, 14.4%), South America (1,390/33,267, 4.2%), Unknown (343/33,267, 1%) and Oceania (100/33,267, 0.3%). ANI analysis confirmed all isolates had values greater than 95% (Supplementary Table 5).

### 3.2 *In silico* analysis: gene presence, sensitivity and specificity of existing targets

A total of five genes were identified and tested for their presence in *N. meningitidis* WGS [*ctrA* (NEIS0055), *sodC* (NEIS1339), *crgA* (NEIS0362), *nspA* (NEIS0612), and *porA* (NEIS1364)]. Gene presence ranged from 98.8% (*ctrA*) to 100% (*nspA*), with primer/probe sensitivities ranging from 0.5% (*crgA*) to 99.7% (*sodC*) and specificities from 99.4% (*sodC*) to 100% (*crgA*). Overall, the best *N. meningitidis* candidate primer sequences targeted *sodC* with a sensitivity of 99.7%, specificity of 99.4%, PPV of 99.6% and NPV of 99.6% closely followed by *porA* (sensitivity: 99.1%, specificity: 99.9%, PPV: 99.8% and NPV: 98.8%) (Table 2).

**TABLE 2:** *In silico* deduced specificity, sensitivity for polymerase chain reaction (PCR) primers and gene presence values for complete coding sequences.

Gene	TP	FP	FN	TN	Sensitivity (%)	Specificity (%)	PPV (%)	NPV (%)	% isolates with the gene	References
***Neisseria meningitidis***										
*ctrA* *	14,055	9	346	10,177	97.6	99.9	99.9	96.7	98.8	Gudza-Mugabe et al., 2015
*sodC* *	14,363	57	38	10,129	99.7	99.4	99.6	99.6	99.9	Diallo et al., 2018
	14,305	57	96	10,129	99.3	99.4	99.6	99.1	–	Thomas et al., 2011
*crgA*	67	0	14,334	10,186	0.5	100	100	41.5	99.8	Taha, 2000
*nspA*	10,814	3	3,587	10,183	75.1	99.9	99.9	74	100	de Filippis et al., 2005
*porA*	7,672	3	6,729	10,183	53.3	99.9	99.9	60.2	99.4	Bennett and Cafferkey, 2006
	14,273	7	128	10,179	99.1	99.9	99.8	98.8	–	Diallo et al., 2018
**Group B streptococci**										
*atr*	67,96	0	1,997	1,181	77.3	100	100	37.1	100	de-Paris et al., 2011
*cfb*	8,728	0	65	1,181	99.3	100	100	94.8	99.8	Carrillo-Ávila et al., 2018
*cylE*	8,680	0	113	1,181	98.7	100	100	91.3	99.7	Bergseng et al., 2007
*dltS*	8,775	0	18	1,181	99.8	100	100	98.5	99.9	Furfaro et al., 2017
*scpB*	0	0	8,793	1,181	0	100	0	11.8	97.6	Elbaradie et al., 2009
*sip*	8,755	0	38	1,181	99.6	100	100	96.9	99.1	Bergh et al., 2004
***Streptococcus pneumoniae***										
*psa*A	32,083	0	1,184	761	96.4	100	100	39.1	99.9	Carvalho et al., 2007
*SP2020*	33,094	1	173	760	99.5	99.9	99.9	81.5	99.8	Tavares et al., 2019
*lyt*A*	32,655	0	612	761	98.2	100	100	55.4	98.0	Carvalho et al., 2007
*ply*	30,666	14	2,601	747	92.2	98.2	99.9	22.3	99.9	Carvalho et al., 2007
*pia*B	23,302	0	9,965	761	70.0	100	100	7.1	98.2	Trzciński et al., 2013
***Haemophilus influenzae***										
*bexA*	273	0	1,691	146	13.9	100	100	7.9	32.9	Wroblewski et al., 2013
*bexB*	158	0	1,806	146	8.0	100	100	7.5	33.5	Davis et al., 2011
*bexD*	148	0	1,816	146	7.5	100	100	7.4	33.5	Lâm et al., 2011
*fucK*	1,892	2	72	144	96.3	98.6	99.9	66.7	97.0	Meyler et al., 2012
hpd*	1,202	3	762	143	61.2	97.9	99.8	15.8	95.5	Maleki et al., 2020
	1,836	3	128	143	93.5	97.9	99.8	52.8	–	Wang et al., 2011
*licA*	1,614	2	350	144	82.2	98.6	99.9	29.1	96.5	Meyler et al., 2012
*ompP2*	842	1	1,122	145	42.9	99.3	99.9	11.4	96.8	Wang et al., 2011
*ompP6*	1,279	55	685	91	65.1	62.3	95.9	11.7	99.8	de Filippis et al., 2016
*pstA*	1,884	2	80	144	95.9	98.6	99.9	64.3	97.0	Coughlan et al., 2015

*Target recommended by the CDC; TP, true positive; FP, false positive; FN, false negative; TN, true negative; PPV, positive predictive value; NPV, negative predictive value.

A total of six genes were identified and tested for their presence in *S. agalactiae* WGS [*atr, cfb* (SAG2043), *cylE* (SAG0669), *dltS* (SAG1791), *scpB* (SAG1236) and *sip* (SAG0032)]. Gene presence ranged from 97.6% (*scpB*) to 100% (*atr*); primer sensitivities ranged from 0% (*scpB*) to 99.8% (*dltS*); specificities and PPV were 100% for all targets tested except *scpB* which had PPV of 0%. NPV ranged from 11.8% (*scpB*) to 98.5% (*dltS*). The best *S. agalactiae* primer sequences targeted *dltS* (with a sensitivity of 99.8%, a specificity of 100%, PPV of 100% and NPV of 98.5%) followed by *sip* (with a sensitivity of 99.6%, specificity of 100%, PPV of 100% and NPV of 96.9%) and *cfb* (sensitivity of 99.3%, specificity of 100%; PPV of 100% and NPV of 94.8%) (Table 2).

A total of five genes were identified and tested for their presence in *S. pneumoniae* WGS [*psaA* (SPNE00983), *SP2020, lytA, ply* (SPNE01149) and *piaB*]. Gene presence ranged from 98.0% (*lytA*) to 99.9% (*psaA* and *ply*), with primer sensitivities ranging from 70.0% (*piaB*) to 99.5% (*SP2020*) and specificities from 98.2% (*ply*) to 100% (*psaA, lytA* and *piaB*). PPVs were 99.9% (*ply* and *SP2020*) and 100% (*psaA, lytA*, and *piaB*) with NPVs ranging from 7.1% (*piaB*) to 81.5% (*SP2020*). In these analyses, the best candidate primers targeted SP2020 (with a sensitivity of 99.5%, specificity of 99.9%, PPV of 99.9% and NPV of 81.5%) followed by *lytA* (with a sensitivity of 98.2%, specificity of 100%, PPV of 100% and NPV of 55.4%) and *psaA* (with a sensitivity of 96.4%, specificity of 100%, PPV of 100% and NPV of 39.1%) (Table 2).

A total of nine genes were identified and tested for their presence in *H. influenzae* WGS [*bexA* (HAEM1156), *bexB* (HAEM1155), *bexD* (HAEM1153), *fucK, hpd* (HAEM0810), *licA* (HAEM1656), *ompP2* (HAEM0191), *ompP6* (HAEM0484) and *pstA* (HAEM1519)]. Gene presence ranged from 32.9% (*bexA*) to 99.8% (*ompP6*), with primer sensitivities from 7.5% (*bexD*) to 96.3% (*fucK*) and specificities from 62.3% (ompP6) to 100% (*bexA, bexB, bexD*). PPVs ranged from 95.9% (*ompP6*) to 100% (*bexA, bexB, bexD*) and NPVs from 7.4% (*bexD*) to 66.7% (*fucK*). Overall, the best candidate genetic target for molecular detection of *H. influenzae* was *fucK* (with a sensitivity of 96.3%, specificity of 98.6%, PPV of 99.9% and NPV of 66.7%), followed by *pstA* (with a sensitivity of 95.9%, specificity of 98.6%, PPV of 99.9% and NPV of 64.3%) and *hpd* (with a sensitivity of 93.5%, specificity of 97.9%, PPV of 99.8% and NPV of 52.8%) (Table 2).

### 3.3 Undetected targets following *in silico* PCR analysis

**FIGURE 1:**
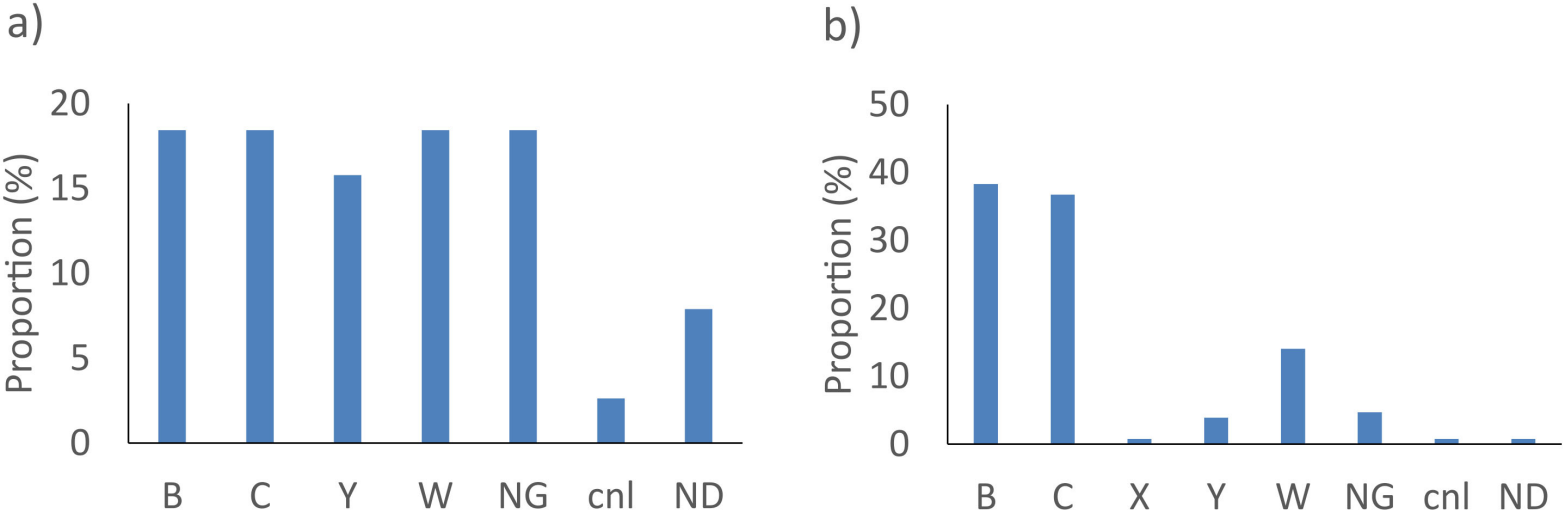
Proportion of genogroup of *N. meningitis* not detected by the best targets: **(a)** *sodC* and **(b)** *porA in silico.* NG, non-groupable; cnl, capsule null locus; ND, not determined.

The gene *sodC* did not detect 38/14401 (0.3%) of the *N. meningitidis* tested. These genomes were either genogroup B, C, W and non-groupable (NG) isolates (7/38, 18.4% each), or genogroup Y (6/38, 15.8%), capsule null (*cnl*) (1/38, 2.6%) or with an undetermined genogroup (3/38, 7.9%) (Figure 1a). The *porA* gene did not detect 128/14401 (0.9%) strains. These were genogroup B isolates (49/128, 38.3%), genogroup C (47/128, 36.7%) or genogroup W (18/128, 14.1%) (Figure 1b).

**FIGURE 2:**
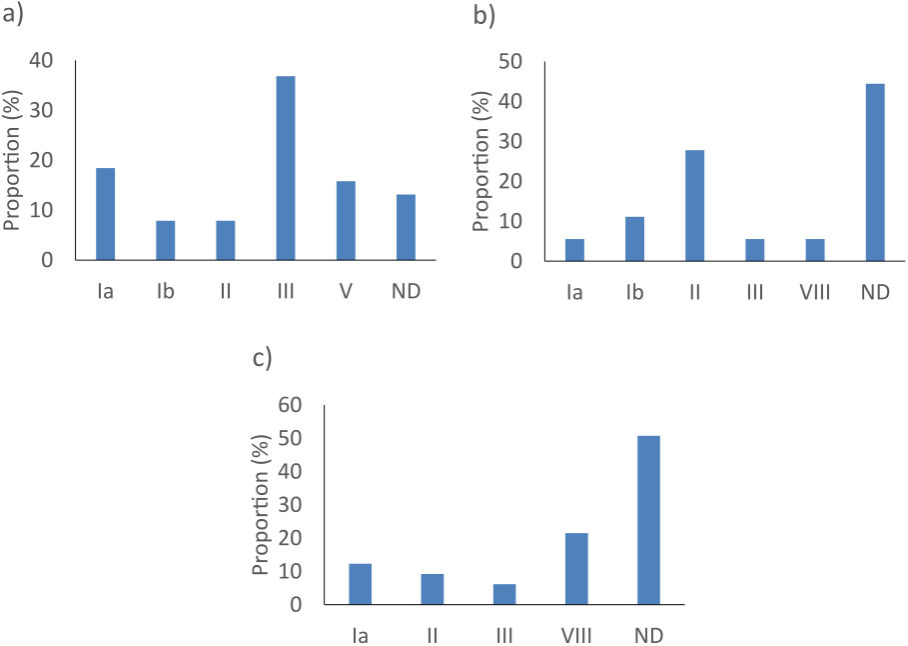
Proportion of serotypes of *Streptococcus agalactiae* not detected by the best targets: **(a)** *sip*, **(b)** *dltS*, and **(c)** *cfb in silico.* ND, not determined.

The *dltS* gene did not detect 18/8793 (0.2%) *S. agalactiae* genomes. These were isolates with an undetermined serotype (8/18, 44.4%) (Figure 2b). The *sip* gene did not detect 38/8793 (0.4%) of the *S. agalactiae* tested. They were predominantly from serotype III isolates (14/38, 36.8%) (Figure 2a). As for *cfb*, it did not detect 65/8793 (0.7%) *S. agalactiae*. These isolates had undetermined serotypes (33/65, 50.8%) (Figure 2c).

**FIGURE 3:**
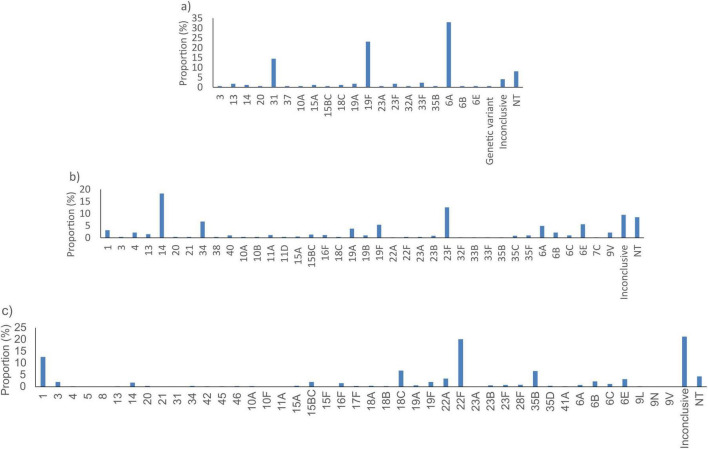
Proportion of serotypes of *S. pneumoniae* not detected by the best targets: **(a)** *SP2020*, **(b)** *lytA*, and **(c)** *psaA in silico*. NT, non-typeable.

*SP2020* did not detect 173/33267 (0.5%) of the *S. pneumoniae* tested. These were predominantly serotypes 6A (57/173, 32.9%), 19F (40/173, 23.1%) and 31 (25/173, 14.5%) (Figure 3a). The *lytA* gene did not detect 612/33267 (1.8%) of the samples tested. These were serotypes 14 (112/173, 18.3%), 23F (77/173, 12.6%) and non-typeable (52/173, 8.5%) and undetermined serotype (58/173, 9.5%) isolates (Figure 3b). The *psaA* gene did not detect 1184/33267 (3.6%) bacterial genomes. These were predominantly from undetermined serotypes (252/1184, 21.3%), serotypes 22F (239/1184, 20.2%) and 1 (150/1184, 12.7%) (Figure 3c).

**FIGURE 4:**
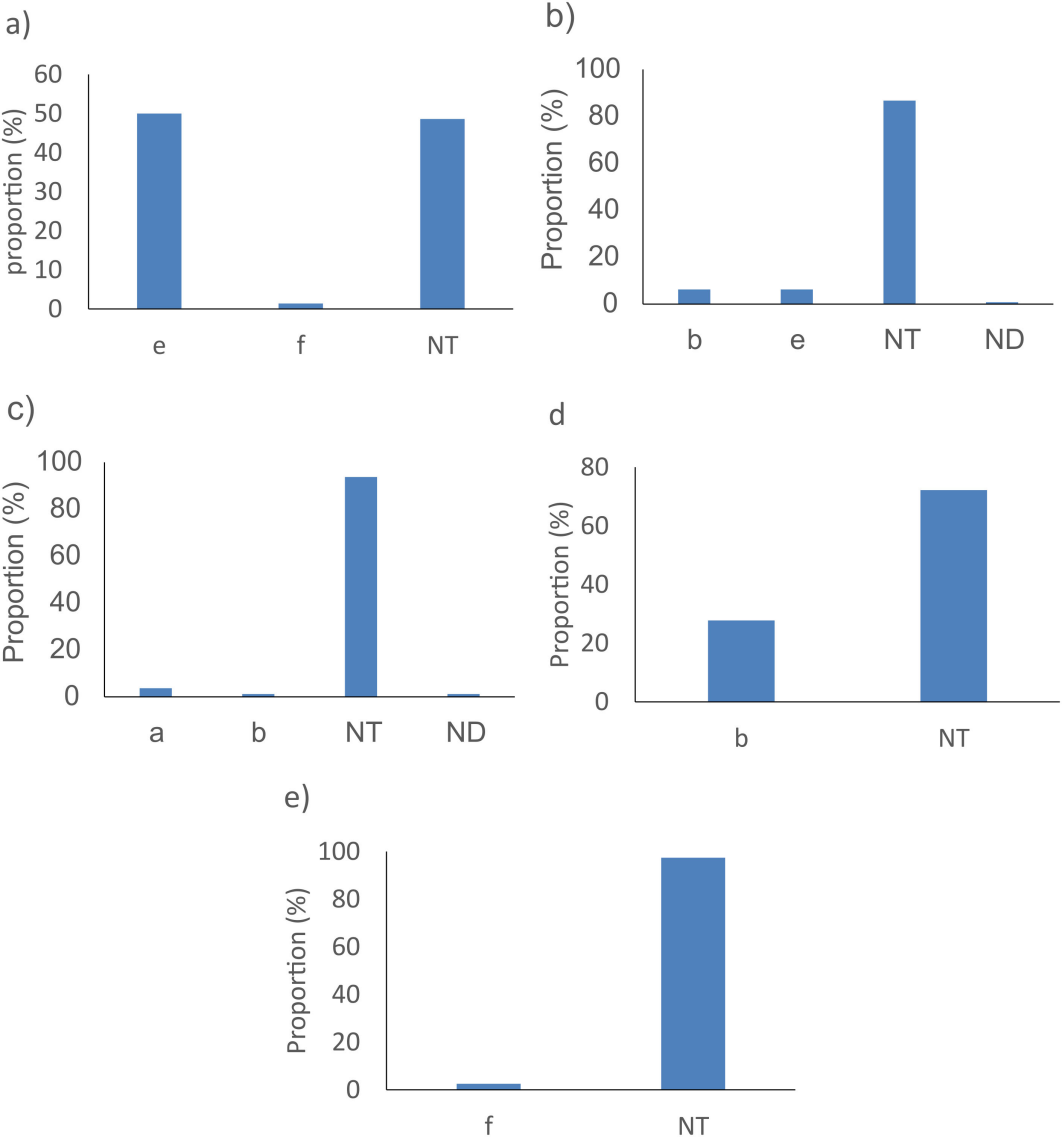
Proportion of serotypes of *H. influenzae* not detected by the best targets: **(a)** *fucK*, **(b)** *hpd*, **(c)** *pstA*, **(d)** *HAEM0428*, and **(e)** *HAEM1183 in silico.* NT, non-typeable, ND, not determined.

The *fucK* gene did not detect 72/1964 (3.7%) *H. influenzae*. These genomes were predominantly from isolates with serotype e (36/72, 50%) and Non-typeable serotype (NT) (35/72, 48.6%) (Figure 4a). The *pstA* gene did not detect 80/1964 (4.1%) bacterial genomes. These were mainly from isolates with Non-typeable serotype (NT) (75/80, 93.8%) (Figure 4c). The *hpd* gene did not detect 128/1964 (6.5%) bacterial genomes. These genomes were predominantly from isolates with Non-typeable serotype (NT) (111/128, 86.7%) (Figure 4b).

### 3.4 Novel targets for detection of *H. influenzae*

Given the sub-optimal *in silico* performance observed for all published *H. influenzae* targets, additional analyses were performed to identify better targets. Comparative genome analyses identified 327 loci that were present in 97% of the *H. influenzae* WGS investigated of which four (*HAEM0428, HAEM1179, HAEM1181*, and *HAEM1183*) were absent or had significantly lower presence in a dataset of 152 other *Haemophilus* species (Supplementary Table 3). *HAEM0428* (*ICMT* gene) encodes protein-S-isoprenylcysteine methyltransferase, *HAEM1179* (*dmsD*) encodes the Tat proofreading chaperone, *HAEM1181* (*dmsC*) encodes Anaerobic dimethyl sulfoxide reductase chain C and *HAEM1183* (*dmsA*) encodes an anaerobic dimethyl sulfoxide reductase chain A. Of these four genes, *HAEM0428* and *HAEM1183* showed better or identical sensitivity and specificities as *fucK*. Indeed, compared to *fucK, HAEM0428* showed similar sensitivity (96.3% vs. 96.3%) but lower specificity (95.9% vs. 98.6%). In contrast, *HAEM1183* showed a better sensitivity (98.0%) and specificity (100%) than *fucK* and *HAEM0428* (Table 3). *In silico* PCR analyses revealed that *HAEM0428* did not detect 72/1964 (3.7%) *H. influenzae*. These isolates were either with Non-typeable serotype (NT) (52/72, 72.2%) or serotype b isolates (20/72, 27.8%) (Figure 4d). *HAEM1183* did not detect 39/1964 (2%) *H. influenzae*. These sequences were in isolates from serotype NT (38/39, 97.4%) (Figure 4e).

**TABLE 3:** Specificity and sensitivity of new assays and *fuc*K for detection of *H. influenzae* obtained *in silico*.

Gene	Sensitivity (%)	Specificity (%)	PPV (%)	NPV (%)
*fucK*	96.3	98.6	99.9	66.7
*HAEM0428*	96.3	95.9	99.7	66.8
*HAEM1179*	95.6	97.3	99.5	62.2
*HAEM1181*	60.5	97.9	99.3	15.6
*HAEM1183*	98.0	100	99.6	77.4

### 3.5 Efficiency of the *in silico* assays by reported isolation source clinical sources

**FIGURE 5:**
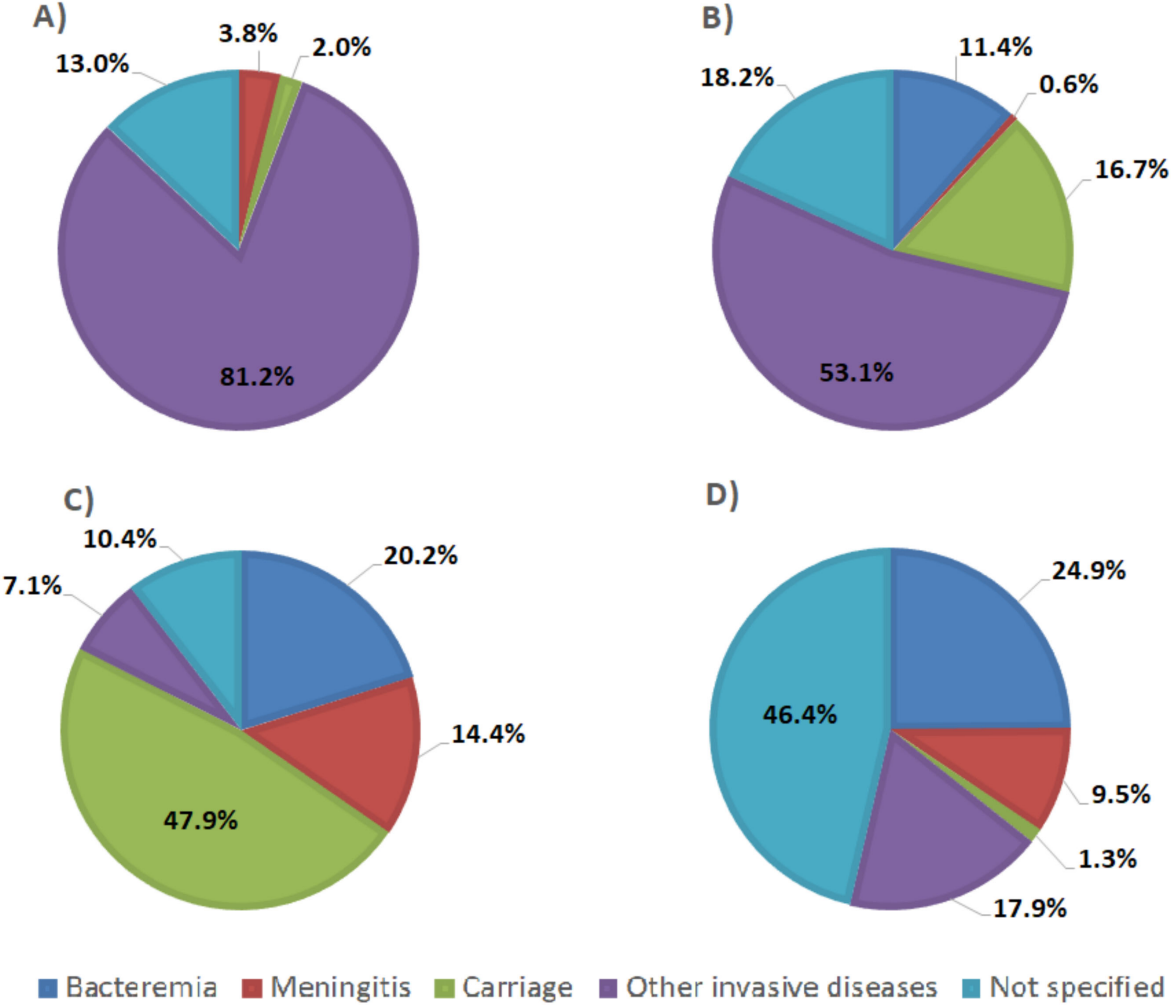
Pie chart representing clinical sources of genomic sequences of **(A)** *N. meningitidis*, **(B)** *Streptococcus agalactiae*, **(C)** *S. pneumoniae*, and **(D)** *H. influenzae* from PubMLST database.

According to the available provenance and phenotype information, 12,241/14,401 (85%) *N. meningitidis* WGS originated from invasive meningococcal disease (IMD), 291/14,401 (2%) were from asymptomatic carriage and 1,869/14,401 (13%) had no information on their isolation source (Figure 5A). The best target genes showed high sensitivity for WGS associated with IMD (92.3%–99.9%) and, specifically, from meningitis cases (94.7%–100%) (Table 4). Indeed, *sodC* detected 12,207/12,241 (99.7%) IMD *N. meningitidis* WGS with 540/542 (99.6%) associated with meningitis only. The *porA* gene detected 12,135/12,241 (99.1%) IMD WGS with 539/542 (99.4%) from meningitis cases only.

**TABLE 4:** Proportion of positives reported as coming from carriage and invasive bacteria including meningitis cases detected by the best targets *in silico*.

Gene	Number of sequences with the target			Number of sequences analyzed			% of sequences from carriage	% of sequences from invasive cases	% of sequences from meningitis cases	References
	Carriage	Invasive cases	Meningitis cases	Carriage	Invasive cases	Meningitis cases				
***Neisseria meningitides***										
*sod*C	291	12,207	540	291	12241	542	100	99.7	99.6	Thomas et al., 2011
*porA*	287	12,135	539	291	12,241	542	98.6	99.1	99.4	Diallo et al., 2018
**Group B streptococci**										
*sip*	1,462	5,696	56	1,467	5725	56	99.7	99.5	100	Bergh et al., 2004
*dltS*	1,464	5,718	56	1,467	5,725	56	99.8	99.9	100	Furfaro et al., 2017
*cfb*	1,464	5,674	56	1,467	5,725	56	99.8	99.1	100	Carrillo-Ávila et al., 2018
***Streptococcus pneumonia***										
*SP2020*	15,798	13,864	4,792	15,926	13,899	4,796	99.2	99.7	99.9	Tavares et al., 2019
*lytA*	15,570	13,690	4,730	15,926	13,899	4,796	97.8	98.5	98.6	Carvalho et al., 2007
*psaA*	15,429	13,318	4,575	15,926	13,899	4,796	96.9	95.8	95.4	Carvalho et al., 2007
***Haemophilus influenza***										
*fucK*	22	1,011	184	26	1,027	187	84.6	98.4	98.4	Meyler et al., 2012
*hpd*	23	948	177	26	1,027	187	88.5	92.3	94.7	Wang et al., 2011
*pstA*	21	964	180	26	1,027	187	80.8	93.9	96.3	Coughlan et al., 2015
*HAEM0428*	22	983	182	26	1,027	187	84.6	95.7	97.3	This study
*HAEM1183*	23	998	184	26	1,027	187	88.5	97.2	98.4	This study

Invasive cases include bacteria from people who have contracted meningitis and other reported diseases. Sequences used for this analysis are hosted on PUBMLST.

A total of 5,725/8,793 (65.1%) *S. agalactiae* WGS were from invasive disease (Figure 5B), 1,467/8,793 (16.7%) were from carriage and 1,601/8,793 (18.2%) were from unspecified sources (Figure 5B). The genetic target, *dltS*, detected 5,718/5,725 (99.9%) of *S. agalactiae* WGS associated with invasive disease and 56/56 (100%) from meningitis cases. The gene *sip* detected 5,696/5,725 (99.5%) WGS associated with invasive disease and 56/56 (100%) from meningitis cases. The *cfb* gene detected 5,674/5,725 (99.1%) invasive disease WGS and 56/56 (100%) from meningitis cases (Table 4).

A total of 13,899/33,267 (41.8%) of *S. pneumoniae* genomes were associated with invasive disease, 15,926/33,267 (47.9%) from carriage and 3,442/33,267 (10.3%) from unspecified sources (Figure 5C). *SP2020* detected 13,864/13,899 (99.7%) of *S. pneumoniae* isolated from invasive cases and 4,792/4,796 (99.9%) from meningitis cases while *lytA* detected 13,690/13,899 (98.5%) of sequences coming from invasive cases and 4,730/4,796 (98.6%) from meningitis cases. The *psaA* gene detected 13,318/13,899 (95.8%) of sequences coming from invasive cases and 4,575/4,796 (95.4%) from meningitis cases (Table 4).

*H. influenzae* genome sequence database included 1,027/1,964 (52.3%) sequences came from invasive diseases, 28/1,964 (1.3%) from carriage and 911/1,964 (46.4%) from unspecified sources (Figure 5D). The *fucK* gene detected 1,011/1,027 (98.4%) of *H. influenzae* from invasive cases and 184/187 (98.4%) from meningitis cases whereas *HAEM1183* detected 998/1,027 (97.2%) of sequences from invasive cases and 184/187 (98.4%) from meningitis cases, and *HAEM 0428* detected 983/1,027 (95.7%) of sequences from invasive cases and 182/187 (97.3%) from meningitis cases. The *pstA* gene detected 964/1,027 (93.9%) of sequences from invasive cases and 180/187 (96.3%) from meningitis cases. The *hpd* gene detected 948/1,027 (92.3%) of sequences from invasive cases and 177/187 (94.7%) from meningitis cases (Table 4).

Sensitivity of targets in sequences from carriage isolates was 291/291 (100%) for *sodC* and 287/291 (98.6%) for *porA* in *N. meningitidis*; 1,462/1,467 (99.7%) for *sip*, 1,464/1,467 (99.8%) for *dltS* and 1,464/1,467 (99.8%) for *cfb* in *S. agalactiae*;15,798/15,926 (99.2%) for *SP2020*, 15,570/15,926 (97.8%) for *lytA* and 15,429/15,926 (96.9%) for *psaA* in *S. pneumoniae*; and 22/26 (84.6%) for *fucK*, 23/26 (88.5%) for *hpd*, 21/26 (80.8%) for *pstA*, 22/26 (84.6%) for *HAEM0428* and 23/26 (88.5%) for *HAEM 1183* in *H. influenzae* (Table 4).

### 3.6 *In vitro* analyses: performance of real-time PCR assays

The genes *sodC* and *porA* were tested for their ability to detect *N. meningitidis*. The two genes showed a sensitivity of 100%, a specificity of 91.7%, a PPV of 72.7% and a NPV of 100% (Table 5 and Supplementary Table 4).

**TABLE 5:** *In vitro* results for real-time polymerase chain reaction (PCR) assays.

Target bacteria	Gene primer set	TP	FP	FN	TN	Sensitivity (%)	Specificity (%)	PPV (%)	NPV (%)
Hi	*hpd*	7	2	0	35	100	94.6	77.8	100
	*pstaA*	7	2	0	35	100	94.6	77.8	100
	*HAEM0428*	7	2	0	35	100	94.6	77.8	100
	*HAEM1183*	7	1	0	36	100	97.3	87.5	100
Nm	*sodC*	8	3	0	33	100	91.7	72.7	100
	*porA*	8	3	0	33	100	91.7	72.7	100
GBS	*sip*	6	0	1	37	85.7	100	100	97.4
	*cfb*	7	0	0	37	100	100	100	100
Sp	*psaA*	8	0	0	36	100	100	100	100
	*SP2020*	8	0	0	36	100	100	100	100

The gene *dltS*, one of the top *S. agalactiae in silico* targets did not perform well in our study using published conditions (Furfaro et al., 2017). This target was therefore not considered further. *cfb* and *sip* had a specificity of 100% and a PPV of 100% each. In addition, *cfb* showed a sensitivity and a NPV of 100% while *sip* showed a sensitivity of 85.7% and a NPV of 97.4% (Table 5 and Supplementary Table 4).

The *lytA* gene target was not tested *in vitro*, due to the presence of *lytA* homologues in pneumococcal prophages (Carvalho et al., 2007). Therefore, *psaA* and *SP2020* were tested for their ability to detect *S. pneumoniae*. These genes had a sensitivity and specificity of 100%. Also, the PPV and NPV of the real-time PCR tests were 100% for *psaA* and *SP2020* (Table 5 and Supplementary Table 4).

The gene, *fucK*, one of the top in silico *H. influenzae* genetic determinants did not work using the reaction conditions described in the original paper (Meyler et al., 2012). This target was therefore not considered further. The remaining targets, *hpd, pstA, HAEM0428* and *HAEM1183*, showed a sensitivity of 100% and a NPV of 100% each. The specificity of *HAEM1183* was 97.3%, *hpd, pstA* and *HAEM0428* were identical (94.6%). PPV was 87.5% for *HAEM1183*, 77.8% for *hpd, pstA* and *HAEM0428* (Table 5 and Supplementary Table 4).

## 4 Discussion

Bacterial meningitis remains a major public health threat, particularly in sub-Saharan Africa due to unpredictable epidemics and the urgent need to improve diagnostic methods for the rapid and accurate detection of the causative pathogens. This study sought to address these challenges using *in silico* approaches with PubMLST, a large nucleotide sequence database, providing a preliminary assessment of the specificity and sensitivity of diagnostic targets to guide *in vitro* validation tests. This approach enables a preliminary assessment of the specificity and sensitivity of diagnostic targets before extensive laboratory testing, significantly reducing the time, effort and costs associated with assay development (Santa Lucia et al., 2020; van Weezep et al., 2019). Promising assays identified *in silico* can then be validated by various laboratories, including those led by citizen scientists, using their available local strains. This collective effort increases the variety of isolates tested and reduces issues related to sample shipments. Additionally, using PubMLST to both select targets and evaluate their performance for *H. influenzae* may introduce a risk of overfitting, since the same dataset informs both steps. PubMLST is the largest and most diverse publicly available database for *H. influenza* genomes. However, it remains important to validate promising targets using independent genomic datasets or clinical isolates to ensure broader applicability and robustness.

Based on our findings, we recommend using *sodC* for *N. meningitidis*, *cfb* for *S. agalactiae*, *SP2020* for *S. pneumoniae*, and *dmsA* for *H. influenzae* due to their high sensitivity, specificity, and consistent prevalence in WGS data. The gene *sodC*, also recommended by WHO/CDC, is highly specific and sensitive for detecting meningococci (Thomas et al., 2011). This gene, which encodes Cu-Zn superoxide dismutase, is ubiquitous in *N. meningitidis* and less likely to undergo antigenic variation due to selective pressure (Thomas et al., 2011). This study also revealed the equivalent efficacy of *porA* (an outer membrane porin). However, rectal and pharyngeal *N. gonorrhoeae* isolates from Australian and Swedish patients have been found to harbor an *N. meningitidis porA* sequence, presumably acquired through horizontal genetic exchange and recombination (Golparian et al., 2012; Whiley et al., 2011). Thus, caution is advised when using *porA* as a target, although detecting *N. gonorrhoeae* in invasive meningococcal cases would be unlikely.

The gene *cfb*, which encodes the extracellular pore-forming toxin (CAMP factor), has been demonstrated as an effective target for GBS detection (Carrillo-Ávila et al., 2018). It is noteworthy that other rtPCR tests such as (i) the Becton Dickinson MAX GBS assay; (ii) the ARIES GBS assay from Luminex Corporation; and (iii) the Xpert GBS LB assay produced by Cepheid Inc. also prioritize *cfb* as the primary target gene (Diallo et al., 2021). However, there is no WHO/CDC recommendation for this gene as GBS is not routinely tested in surveillance.

Despite *lytA* (the major autolysin of pneumococcus) being widely recommended by WHO/CDC and routinely used in surveillance of *S. pneumoniae* (Satzke et al., 2013), concerns arise due to its homologs in closely related *Streptococcus* species (Tavares et al., 2019), potentially increasing the false positivity rate (Ganaie et al., 2021; Llull et al., 2006; Simões et al., 2016). Furthermore, the work of Martín-Galiano and García (2021) revealed that pneumococcal prophages harbor *lytA*-like genes homologous to *S. pneumoniae lytA*, and that there were recombination events between the pneumococcal and phage *lytA* homologs, further questioning its reliability. In contrast, *SP2020* (a putative transcriptional regulator gene of the GntR family) has been shown to be a better target than *lytA* for *S. pneumoniae* diagnosis, consistent with the findings of Tavares et al. (2019). In this study, *lytA* was included in the *in silico* analysis to enable direct comparison with *SP2020* due to its common use. Given its limitations, *lytA* was not evaluated further *in vitro*. The results support the use of *SP2020* as a preferred diagnostic target.

The gene *fucK* (gene encoding fuculokinase) demonstrated the best overall performance *in silico* for the identification of *H. influenzae*. However, this target gene performed poorly in comparison to the ones used for the three other pathogens, suggesting a need for improvement of *H. influenzae* diagnostic determinants. Furthermore, more than 1% of non-*Hi* sequences had the *fucK* gene and this gene did not detect all *H. influenzae*, as reported by de Gier et al. (2015). We were unable to amplify *fucK in vitro* using the published conditions (Meyler et al., 2012). Therefore, although *in silico* analyses suggest that it is a promising target, we cannot confidently assert that it is the best target without experimental validation. It would thus be beneficial to optimize the existing primers or design new ones. In contrast, *dmsA* performed better than *hpd*, the gene recommended by WHO/CDC (Wang et al., 2011), suggesting its efficacy for *H. influenzae* identification. The *dmsA* gene is required for fitness of *H. influenzae* (Dhouib et al., 2021) and appears to be the most efficient test for the identification of *H. influenzae* in our study. Nasreen et al. (2024) showed that the DmsABC complex protects *H. influenzae* against oxidative stress, particularly from host-derived hypochlorite. Expression of *dmsA* increases under such stress, and its deletion impairs bacterial survival and intracellular persistence. These findings suggest that *dmsA* contributes to both stress adaptation and host interaction.

The PubMLST database includes isolates from various sources, some known to be from asymptomatic carriage and others from unspecified sources. Our assays are able to detect isolates from these different sources. Additional analysis was performed to evaluate the efficacy of the targets for bacteria isolated from cases of invasive disease and/or meningitis. All four targets exhibited high sensitivity for the target bacteria from invasive disease (97.2%–99.7%) and for bacteria from meningitis specimens (98.4%–100%). Further evaluation with more specimens from invasive diseases from diverse geographical regions, which was beyond the scope of the present work, would be useful to confirm the results presented here. Although some bacterial variants are currently more prevalent than others, the inclusion of non-invasive isolates in the different test panels remains important. Indeed, these strains have no intrinsic factors that prevent them from causing disease, and also need to be monitored because they can acquire virulent genes especially as they are exposed to new vaccine pressures such as for *N. meningitidis* and potentially for *S. agalactiae*. This has been shown with the non-virulent *N. meningitidis* carriage strain that acquired both a serogroup C capsule and the filamentous bacteriophage MDAΦ, which has been shown to enhance colonization of nasopharyngeal epithelial cells, increasing virulence, and leading to epidemics first reported in 2013 in the Tambuwal area of Nigeria, with the strain spreading to different regions of Niger (Brynildsrud et al., 2018). Similarly, in *S. pneumoniae*, non-encapsulated strains (NESp) typically cause non-invasive pneumococcal diseases. However, NESp strains have recently been identified as causative agents of invasive disease. Bradshaw et al. (2020) demonstrated that NESp are highly transformable, capable of acquiring large DNA segments that increase their persistence and virulence during invasive disease. Group B streptococci (GBS), commensals of the vagina and gastrointestinal tract, can become invasive, particularly in newborns, through GBS adaptation to environmental changes under the control of the CovRS two-component regulatory system (Patras et al., 2013). Genomic mutations, including those affecting capsule synthesis regulator (CovR), also appear to influence the transition of GBS from a commensal state to a pathogen and its ability to persist in mothers before and after delivery (Shabayek and Spellerberg, 2018). The assays failed to detect the sequences of the target genes analyzed in isolates belonging to certain serotypes or capsules *in silico* due to complete or partial deletion of these genes (Khatami et al., 2018; Whyte et al., 2020) in some strains and due to the stringent conditions applied (no mismatches allowed in the primers). This issue needs to be monitored in real life, as some missed genotypes, such as serogroups B and C in *N. meningitidis*, serotypes Ia, Ib, III, and V in *S. agalactiae*, serotypes 4, 14, 7F, 9V, and 18C in *S. pneumoniae*, and non-typeable *H. influenzae* (NTHi), can cause disease (Lambertsen et al., 2010; Levy et al., 2010; Moreno et al., 2020; Resman et al., 2011; Sleeman et al., 2006; Tazi et al., 2011).

Our *in silico* analysis was conducted using WGS databases, which only include culturable bacteria. All available genome sequences are derived from cultured bacteria, which can limit the diversity captured, especially for strains that are difficult to grow. This approach may introduce bias as the need to culture pathogens for whole genome sequencing (WGS) limits the representativeness of the data. Primer design depends on the available sequence data, which is currently mostly from cultured bacteria. As a result, the diversity of uncultivable strains may be overlooked and PCR may lack the sensitivity to detect them. One solution to improve representativeness would be to use culture-independent sequencing methods, such as metagenomics, which can explore a wider bacterial diversity without relying on specific primers. These allow detection of both culturable and unculturable strains and may reveal additional genetic targets for more sensitive molecular diagnostics. Furthermore, according to the WHO report, laboratory data from weeks 1 to 30 of 2024 (January 1 to July 28) indicated that 4,926 cerebrospinal fluid (CSF) samples tested by PCR out of 7,468 suspected cases were negative, despite strong clinical suspicions of meningitis (World Health Organization [WHO], 2024). These results suggest either the presence of other pathogens that current tests do not detect, or a lack of sensitivity in the current diagnostic methods. In this context, our study is particularly relevant. By improving diagnostic tools, we aim to enhance the detection capacity of the four most virulent meningitis pathogens. However, this approach must be expanded to identify other genes for diagnosing additional pathogens responsible for meningitis, such as viruses or other infectious agents known to be difficult to detect with current methods. WHO data highlights the critical need to develop more sensitive diagnostic tests adapted to the contexts of low- and middle-income countries (LMICs). Given the healthcare challenges posed by the burden of infectious diseases in these regions, implementing tools suited to local conditions is essential.

In conclusion, the genes *sodC, cfb, SP2020*, and *dmsA* have allowed for the *in silico* identification of *N. meningitidis*, *S. agalactiae, S. pneumoniae*, and *H. influenzae*, respectively, from various clinical sources, including invasive cases, and specifically in cases reported clinically as meningitis, and have shown promising results *in vitro* despite the limited number of samples tested. The diagnostic measures should nevertheless be interpreted with caution given the absence of confidence intervals and formal statistical testing. These genes thus have the potential to significantly enhance the precision of molecular diagnostics for meningitis. However, laboratory confirmation with a larger number of samples, including patient samples such as CSF or blood, remain necessary. Additionally, the performance of these targets in cases of co-infection or samples with low pathogen loads was not assessed in this study due to limited data. Future work should evaluate diagnostic accuracy under these conditions to ensure reliability in diverse clinical scenarios. The *in silico* approach, utilizing extensive WGS databases such as PubMLST, combined with the *in vitro* approach, enables efficient and cost-effective preliminary evaluation of diagnostic targets. This can be particularly beneficial in situations characterized by variability in etiological agents and potential changes in their relative prevalence due to collective immunity induced by vaccines, especially in resource-constrained environments.

## Data Availability

The datasets presented in this study can be found in online repositories. The names of the repository/repositories and accession number(s) can be found in the article/Supplementary material.

## Footnotes

1
https://pubmlst.org

2
https://pubmlst.org/species-id

3
https://pubmlst.org/organisms/haemophilus-influenzae
