# Supplementary material for: In silico and in vitro analyses for the improved diagnosis of bacterial meningitis
Source: Front Microbiol. 2025 Sep 26;16:1655490. doi: 10.3389/fmicb.2025.1655490 (PMC12511035; doi:10.3389/fmicb.2025.1655490)
Supplement: Supplementary file 1 [file Table_1.docx]

Supplementary data


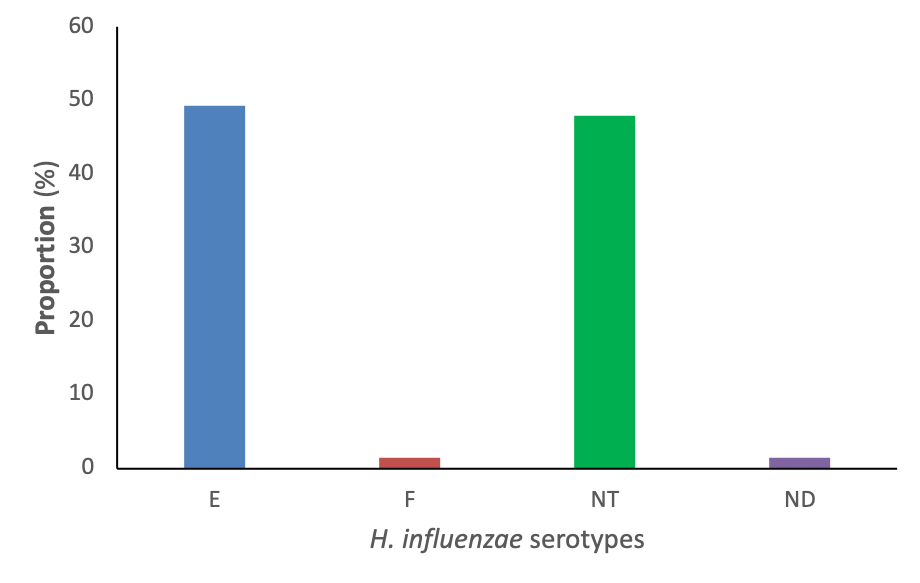


**c**

Table S1: Primer sequences and references for *in silico* analysis

| **Gene** | **Primer name** | **Sequence** | **Reference** | **PubMLST loci or NCBI number** |
| --- | --- | --- | --- | --- |
| *Haemophilus influenzae* | | | | |
| *bexA* | BexA-F | TCTTGGCCGTTAGCTTTTAGTGG | (Wroblewski et al., 2013) | HAEM1156 |
|  | BexA-R | CATAAAGATAATCGCCCAATTCAGA |  |  |
| *bexB* | HI-1 | GGTGATTAACGCGTTGCTTATGCG | (Davis et al., 2011) | HAEM1155 |
|  | HI-2 | TTGTGCCTGTGCTGGAAGGTTATG |  |  |
| *bexD* | TTL2 | AATTATTGTTAGCAATTTTAACTAC | (Lâm et al., 2011) | HAEM1153 |
|  | TTL5 | GCGTTAAGCTTGTATTAAACTG |  |  |
| *fucK* | Forward | ATGGCGGGAACATCAATGA | (Meyler et al., 2012) | fucK |
|  | Reverse | ACGCATAGGAGGGAAATGGTT |  |  |
| *hpd* | F | GATGGCTTGACTGATGTTGC | (Maleki et al., 2020) | HAEM0810 |
|  | R | TTTGCCATCTTTGGTTTCAA |  |  |
|  | hpdF822 | GGTTAAATATGCCGATGGTGTTG | (Wang et al., 2011) |  |
|  | hpdR952 | TGCATCTTTACGCACGGTGTA |  |  |
| *licA* | Forward | TCATTTATCGAAAGAAGCTGCTGAT | (Meyler et al., 2012) | HAEM1656 |
|  | Reverse | CCGTTTGTGAGCGATTTGAA |  |  |
| *ompP2* | P2Fw | GGTGCATTCGCAGCTTCAG | (Wang et al., 2011) | HAEM0191 |
|  | P2Rv | GATTGCGTAATGCACCGTGTT |  |  |
| *ompP6* | HiP6-F | ACTTTTGGCGGTTACTCTGT | (de Filippis et al., 2016) | HAEM0484 |
|  | HiP6-R | TGTGCCTAATTTACCAGCAT |  |  |
| *pstA* | pstA P1 | CGTTTCGCACAAATTACC | (Coughlan et al., 2015) | HAEM1519 |
|  | pstA P2 | GTGCGTACCACGATAGG |  |  |
| *HAEM*  *0428* | HAEM0428-F | TGCCTGTATTTTAGCGATCCG | This study | HAEM  0428 |
|  | HAEM0428-R | ATTAGCCTCAATGATCGCCG |  |  |
| *HAEM*  *1179* | HAEM1179-F | CCTCCCTTTTCAGCACGAGA |  | HAEM  1179 |
|  | HAEM1179-R | TCTGCGCATTTACCACACAA |  |  |
| *HAEM*  *1181* | HAEM1181-F | TGGATTAACATTGAGCTATGCGT |  | HAEM  1181 |
|  | HAEM1181-R | GAGGTCAGCGGCATTTTGAA |  |  |
| HAEM  1183 | HAEM1183-F | TATGGTACGGGAACACTCGG |  | HAEM  1183 |
|  | HAEM1183-R | ATTTCCCAATGCCCAACCAC |  |  |
| Group B *Streptococcus* | | | | |
| *atr* | F | CAACGATTCTCTCAGCTTTGTTAA | (de-Paris et al., 2011) | atr |
|  | R | TAAGAAATCTCTTGTGCGGATTTC |  |  |
| *cfb* | F | GAAACATTGATTGCCCAGC | (Carrillo-Ávila et al., 2018) | SAG2043 |
|  | R | AGGAAGATTTATCGCACCTG |  |  |
| *cylE* | Primer 1 | TGACATTTACAAGTGACGAAG | (Bergseng et al., 2007) | SAG0669 |
|  | Primer 2 | TTGCCAGGAGGAGAATAGGA |  |  |
| *dltS* | dltSF | CCTTATGGCGTTCCACGATT | (Furfaro et al., 2017) | SAG1791 |
|  | dltSR | ATCATGCAGATTCTCTCAGTTTTGG |  |  |
| *scpB* | scpB-F | ACAACGGAAGGCGCTACTGTTC | (Elbaradie et al., 2009) | SAG1236 |
|  | scpB-R | ACCTGGTGTTTGACCTGAACTA |  |  |
| *sip* | sip-F | ATCCTGAGACAACACTGACA | (Bergh et al., 2004) | SAG0032 |
|  | sip-R | TTGCTGGTGTTTCTATTTTCA |  |  |
| *Neisseria* *meningitidis* | | | | |
| *ctrA* | F753 | TGTGTTCCGCTATACGCCATT | (Gudza-Mugabe et al., 2015) | NEIS0055 |
|  | R846 | GCCATATTCACACGATATACC |  |  |
| *sodC* | sodC-F2 | GCGGTTAGTGCAGTATGTTCAG | (Diallo et al., 2018) | NEIS1339 |
|  | sodC-R2 | TAATCACGCCACATGCCATA |  |  |
|  | fwd 351 | GCACACTTAGGTGATTTACCTGCAT | (Thomas et al., 2011) |  |
|  | Rev 478 | CCACCCGTGTGGATCATAATAGA |  |  |
| *crgA* | crgA-F | GCTGGCGCCGCTGGCAACAAAATTC | (Taha, 2000) | NEIS0362 |
|  | crgA-R | CTTCTGCAGATTGCGGCGTGCCGT |  |  |
| *nspA* | nspA-1 | AGCACTTGCCACACTGATTG | (de Filippis et al., 2005) | NEIS0612 |
|  | nspA-2 | GGAACGGACGTTTTTGACAG |  |  |
| *porA* | porA_fwd_1 | GCCGGCGTTGATTATGATTT | (Diallo et al., 2018) | NEIS1364 |
|  | porA_rev_1 | AGTTGCCGATGCCGGTATT |  |  |
|  | porA 2F | GCGGTTTTGCCGGGAACTAT | (Bennett and Cafferkey, 2006) |  |
|  | porA 15R | AGTGGCGGCAATTTCGGTCGTACT |  |  |
| *Streptococcus pneumoniae* | | | | |
| *psaA* | Forward | GCCCTAATAAATTGGAGGATCTAATGA | (Carvalho et al., 2007) | SPNE00983 |
|  | Reverse | GACCAGAAGTTGTATCTTTTTTTCCG |  |  |
| *SP2020* | SP_2020_F | TAAACAGTTTGCCTGTAGTCG | (Tavares et al., 2019) | AE005672.3, nt 1925563 to 1926291 |
|  | SP_2020_R | CCCGGATATCTCTTTCTGGA |  |  |
| *lytA* | lytA-CDC forward | ACGCAATCTAGCAGATGAAGCA | (Carvalho et al., 2007) | AE005672.3, nt 1840405 to 1841361 |
|  | lytA-CDC reverse | TCGTGCGTTTTAATTCCAGCT |  |  |
| *ply* | ply-CDC forward | GCTTATGGGCGCCAAGTCTA | (Carvalho et al., 2007) | SPNE01149 |
|  | ply-CDC reverse | CAAAGCTTCAAAAGCAGCCTCTA |  |  |
| *piaB* | PiaF | CATTGGTGGCTTAGTA AGTGCAA | (Trzciński et al., 2013) | AE005672.3, nt 974409 to 975428 |
|  | PiaR | TACTAACACAAGTTCCTGATAAGGCAAGT |  |  |
