# Supplementary material for: In silico and in vitro analyses for the improved diagnosis of bacterial meningitis
Source: Front Microbiol. 2025 Sep 26;16:1655490. doi: 10.3389/fmicb.2025.1655490 (PMC12511035; doi:10.3389/fmicb.2025.1655490)
Supplement: Supplementary file 2 [file Table_2.docx]

Supplementary data


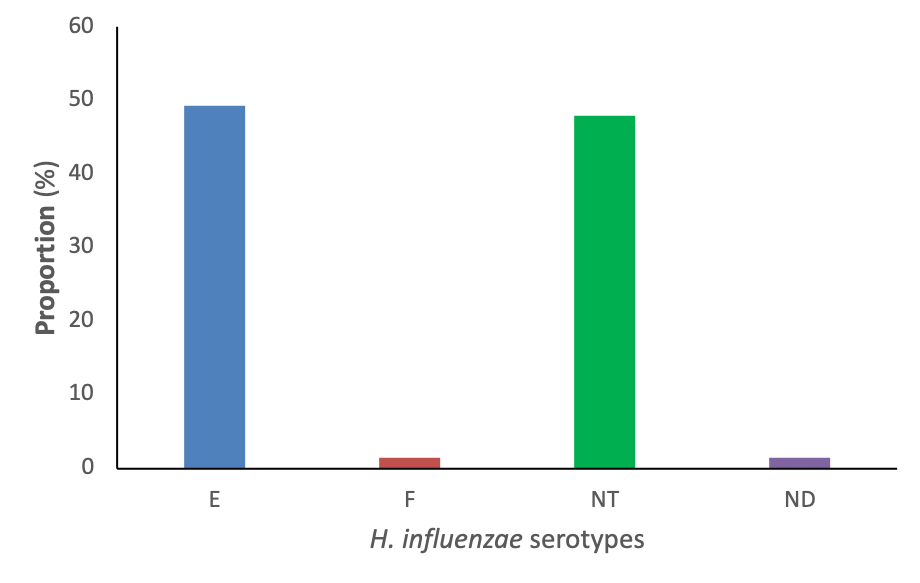


**c**

Table S2: Representative bacterial isolate panel

| **Strains** | **ID** | **Clinical source** | **Country** |
| --- | --- | --- | --- |
| *Haemophilus influenzae a* | 66643 | Blood culture | South Africa |
| *Haemophilus influenzae b* | 67405 | CSF | South Africa |
| *Haemophilus influenzae c* | 67657 | Blood Culture | South Africa |
| *Haemophilus influenzae d* | 65424 | Blood culture | South Africa |
| *Haemophilus influenzae e* | 61595 | Blood culture | South Africa |
| *Haemophilus influenzae f* | 67954 | CSF | South Africa |
| *Haemophilus haemolyticus* | QAF GSK | Control strain | South Africa |
| *Neisseria meningitidis A* | ATCC13077 | Control strain | South Africa |
| *Neisseria meningitidis B* | 61370 | Blood culture | South Africa |
| *Neisseria meningitidis C* | 61635 | Blood culture | South Africa |
| *Neisseria meningitidis X* | 46414 | Blood culture | South Africa |
| *Neisseria meningitidis W* | 65322 | Blood culture | South Africa |
| *Neisseria meningitidis Y* | 61697 | Blood culture | South Africa |
| *Neisseria lactamica* | ATCC23970 | Control strain | South Africa |
| *Neisseria gonorrhoeae* | WHO | Control strain | South Africa |
| *Streptococcus agalactiae Ia* | 62983 | Blood culture | South Africa |
| *Streptococcus agalactiae Ib* | 62968 | Blood culture | South Africa |
| *Streptococcus agalactiae II* | 62378 | Tissue | South Africa |
| *Streptococcus agalactiae III* | 63709 | Blood culture | South Africa |
| *Streptococcus agalactiae IV* | 63381 | CSF | South Africa |
| *Streptococcus agalactiae V* | 63735 | Blood culture | South Africa |
| *Streptococcus pneumoniae 6A* | 65954 | Pleural fluid | South Africa |
| *Streptococcus pneumoniae 6B* | 66773 | Blood culture | South Africa |
| *Streptococcus pneumoniae 1* | 60105 | Blood culture | South Africa |
| *Streptococcus pneumoniae 12F* | 67999 | Blood culture | South Africa |
| *Streptococcus pneumoniae 14* | 67044 | Blood Culture | South Africa |
| *Streptococcus pneumoniae 19F* | 68003 | Blood culture | South Africa |
| *Streptococcus pneumoniae 23F* | 67951 | CSF | South Africa |
| *Neisseria meningitidis* | 04-0005-S3 Oro | Oropharyngeal sample | Côte d'Ivoire |
| *Neisseria meningitidis* | 04-0005-S3 Sal | Saliva sample | Côte d'Ivoire |
| *Neisseria lactamica* | 04-0036-1 | Oropharyngeal sample | Côte d'Ivoire |
| *Neisseria lactamica* | 04-0036-3 | Oropharyngeal sample | Côte d'Ivoire |
| *Neisseria bergeri* | 04-0006-3 | Oropharyngeal sample | Côte d'Ivoire |
| *Neisseria* *bergeri* | 04-0020-3-Oro | Oropharyngeal sample | Côte d'Ivoire |
| *Neisseria* *bergeri* | 04-0020-3-Sal | Saliva sample | Côte d'Ivoire |
| *Moraxella catarrhalis* | 03-0025-4 | Oropharyngeal sample | Côte d'Ivoire |
| *Moraxella catarrhalis* | 03-0028-5 | Oropharyngeal sample | Côte d'Ivoire |
| *Haemophilus influenzae* | NCTC 8143 | Control strain | United Kingdom |
| *Haemophilus haemolyticus* | NCTC 10659 | Control strain | United Kingdom |
| *Haemophilus aegyptius* | NCTC 8502 | Control strain | USA |
| *Neisseria lactamica* | NCTC 10617 | Control strain | USA |
| *Streptococcus agalactiae* | NCTC 8181 | Control strain | United Kingdom |
| *Streptococcus pneumoniae* | NCTC 7465 | Control strain | USA |
| *Streptococcus mitis* | NCTC 12261 | Control strain | Denmark |
| The ID column refers to the code assigned by the laboratory or organisation that provided the strain. | | | |
