# Supplementary material for: In silico and in vitro analyses for the improved diagnosis of bacterial meningitis
Source: Front Microbiol. 2025 Sep 26;16:1655490. doi: 10.3389/fmicb.2025.1655490 (PMC12511035; doi:10.3389/fmicb.2025.1655490)
Supplement: Supplementary file 3 [file Table_3.docx]

Supplementary data

Table S3: *In silico* genome comparator analysis performed with a core threshold of 97% in *Haemophilus influenzae* database

| **Gene** | **% Hi with the gene** | **% non-Hi with the gene** |
| --- | --- | --- |
| 212 HAEM locus between HAEM0005 and HAEM1842 | 97.0 - 98.9 | 32.88 – 100 |
| HAEM 0428 | 98.6 | 7.38 |
| HAEM1179 | 98.8 | 13.42 |
| HAEM1181 | 98.3 | 14.76 |
| HAEM1183 | 97.0 | 17.44 |
| *Adk* | 99.9 | 100 |
| *atpG* | 99.6 | 100 |
| *frdB* | 99.3 | 100 |
| *ftsI* | 99.3 | 100 |
| 103 HAEM locus between HAEM0001 and HAEM1867 | 99.0 - 99.9 | 36.91 – 100 |
| *Mdh* | 99.7 | 98.66 |
| *Pgi* | 99.2 | 100 |
| *recA* | 99.3 | 100 |
| *fucK* | 97.0 | 26.17 |
| The analysis was carried out using 1974 *Haemophilus influenzae* (Hi) sequences used in this study and hosted on PubMLST. 317 genes were found with a minimum threshold of 97%. These genes were then evaluated for their presence in 152 sequences of other *Haemophilus* (non-Hi). | | |
