# Supplementary material for: In silico and in vitro analyses for the improved diagnosis of bacterial meningitis
Source: Front Microbiol. 2025 Sep 26;16:1655490. doi: 10.3389/fmicb.2025.1655490 (PMC12511035; doi:10.3389/fmicb.2025.1655490)
Supplement: Supplementary file 4 [file Table_4.docx]

Supplementary data

Table S4: Ct values of Real-time PCR assays from *in vitro* analysis

| DNA sample | Ct value | | | | | | | | | |
| --- | --- | --- | --- | --- | --- | --- | --- | --- | --- | --- |
|  | *SodC* | *porA* | *sip* | *cfb* | *SP2020* | *psaA* | *pstA* | *hpd* | *HAEM0428* | *HAEM1183* |
| Hi NCTC 8143 | - | - | - | - | 41.25 | - | 24.57 | 23.44 | 22.62 | 25.08 |
| Hia-66643 | - | - | 39.22 | 39.2 | - | - | 21.6 | 17.19 | 18.9 | 21.35 |
| Hib-67405 | - | - | - | - | - | - | 22.87 | 19.86 | 21.24 | 22.49 |
| Hic-67657 | - | - | - | - | 43.49 | - | 18.57 | 14.79 | 16.42 | 19.6 |
| Hid-65424 | - | - | - | - | - | - | 20.24 | 17.31 | 17.22 | 31.25 |
| Hie-61595 | 41.81 | 37.65 | - | - | 38.51 | - | 19.43 | 17.01 | 18.04 | 20.8 |
| Hif-67954 | - | - | 39.13 | 37.59 | - | - | 19.22 | 16.34 | 16.58 | 22.96 |
| *H. haemolyticus* QAF GSK | 40 | - | - | - | - | - | - | - | - | - |
| *H. haemolyticus* NCTC 10659 | - | - | - | - | - | - | 40.13 | 39.85 | - | 40.52 |
| *H. aegyptius* NCTC 8502 | - | - | - | - | - | - | 34.54 | 33.77 | 29.75 | 35.87 |
| NmA- ATCC13077 | 22.4 | 22.03 | - | - | - | 42.12 | - | - | - | - |
| NmB-61370 | 21.36 | 20.47 | - | - | - | 37.61 | - | - | - | - |
| NmC-61635 | 20.64 | 19.87 | - | - | - | 39.21 | - | - | - | - |
| NmW-65322 | 18.56 | 17.17 | 43.24 | - | - | 42.47 | - | - | - | - |
| NmX-46414 | 20.07 | 18.45 | - | - | 41.19 | - | - | - | - | - |
| NmY-61697 | 17.67 | 16.88 | - | - | - | 35.96 | - | - | - | - |
| Nm-04-0005-S3 Oro | 16.08 | 15.34 | - | - | - | - | - | - | - | - |
| Nm-04-0005-S3 Sal | 13.58 | 14.08 | - | - | - | - | - | - | 38.14 | - |
| *N. lactamica* ATCC23970 | 35.63 | 33.52 | 41.81 | 39.02 | 42.72 | 37.03 | 36.01 | 36.31 | - | - |
| *N. lactamica*-CSRS | 37.84 | - | - | - | - | - | - | - | - | - |
| *N. lactamica*-CSRS | 38.22 | 36.98 | - | - | - | 38 | - | - | - | - |
| *N. lactamica* NCTC 10617 | - | - | 43.44 | 41.08 | 40.05 | 39.3 | 40.46 | - | 31.03 | 34.38 |
| *N. gonorrhée* WHO | 40.07 | - | - | 41.72 | - | - | 41.6 | - | - | - |
| *Neisseria* *bergeri* 04-0006-3 | 16.75 | 32.6 | - | - | - | 41.76 | - | - | - | - |
| *Neisseria* *bergeri* 04-0020-3-Oro | 18.66 | 37.11 | - | - | - | - | - | - | - | - |
| *Neisseria* *bergeri* 04-0020-3-Sal | 33.44 | 34.49 | - | - | - | 44.16 | - | - | - | - |
| GBS NCTC 8181 | 40.67 | - | 19.48 | 16.69 | 36.7 | 35.37 | 32.48 | 29.59 | 45.38 | 45.89 |
| GBSIa-62983 | - | - | 21.49 | 22 | 42 | 36.31 | - | - | - | 42.5 |
| GBSIb-62968 | - | - | - | 19.22 | - | - | - | - | - | - |
| GBSII-62378 | - | - | 19.04 | 19.15 | - | - | 42.49 | 41.25 | - | - |
| GBSIII-63709 | - | - | 18.68 | 17.99 | - | - | - | - | - | - |
| GBSIV-63381 | - | - | 18.75 | 17.83 | - | - | - | - | - | - |
| GBSV-63735 | - | - | 20.4 | 21.33 | 41.39 | 38.09 | 40.69 | - | - | - |
| Sp NCTC 7465 | - | - | 38.76 | 39.62 | 21.82 | 20.35 | 38.41 | 37.31 | - | 39.57 |
| Sp6A-65954 | - | - | - | - | 20.26 | 18.63 | 39.09 | - | 65954- | - |
| Sp6B-66773 | - | - | - | - | 25.06 | 21.46 | 38.78 | - | - | - |
| Sp1-60105 | - | - | - | - | 21.36 | 21.06 | - | - | - | - |
| Sp12F-67999 | - | 39.27 | 39.19 | - | 21.17 | 17.86 | 43.43 | - | - | - |
| Sp14-67044 | - | - | 40.69 | 39.91 | 16.24 | 14.32 | - | - | - | - |
| Sp19F-68003 | - | - | - | - | 17.37 | 15.53 | - | - | - | - |
| Sp23F-67951 | - | - | - | - | 21.29 | 19.34 | - | - | - | - |
| *S. mitis* NCTC 12261 | - | - | - | 43.04 | - | - | - | 36.2 | 38.57 | 36.13 |
| *Moraxella catarrhalis*-03-0025-4 | 38.3 | 38.76 | - | - | - | - | - | - | - | - |
| *Moraxella catarrhalis*-03-0028-5 | 35.2 | 38.4 | - | - | - | - | - | - | - | - |
| Water, Molecular Biology Grade | - | - | - | - | - | - | - | - | - | - |
| Ct <35: positive result; Ct ≥ 35: negative result; -: negative result with Ct >50; NCTC, ATCC strains, *N. gonorrhée* WHO and *H. haemolyticus* QAF GSK were control strains; Oro and Sal mean that strains were isolated from oropharyngeal and saliva samples respectively. The strains were from blood culture, CSF, pleural fluid, patient tissue, oropharyngeal and saliva samples. | | | | | | | | | | |
